# Dental Fluorosis according to Birth Cohort and Fluoride Markers in an Endemic Region of Colombia

**DOI:** 10.1155/2021/6662940

**Published:** 2021-03-08

**Authors:** Alexandra Saldarriaga, Manuel Restrepo, Diego F. Rojas-Gualdrón, Thamyris de Souza Carvalho, Marilia Afonso Rabelo Buzalaf, Lourdes Santos-Pinto, Fabiano Jeremias

**Affiliations:** ^1^Graduate Program in Dental Science, São Paulo State University (UNESP), School of Dentistry, Araraquara, São Paulo, Brazil; ^2^School of Dentistry, CES University, Medellín, Colombia; ^3^School of Medicine, CES University, Medellin, Colombia; ^4^Department of Biological Sciences, University of São Paulo (USP), Bauru School of Dentistry, Bauru, São Paulo, Brazil; ^5^Department of Morphology and Orthodontics and Pediatric Dentistry, São Paulo State University (UNESP), School of Dentistry, Araraquara, São Paulo, Brazil

## Abstract

**Objectives:**

To analyze changes in the dental fluorosis (DF) incidence according to a birth cohort and explore current exposure to DF in a case series.

**Methods:**

Repeated cross-sectional study of two periods: 2015 and 2018. Two standardized examiners registered DF using the Thylstrup-Fejerskov index in permanent teeth of children aged 7–18 years. Period and birth cohort frequencies were estimated by a generalized linear model, binomial family, and logarithmic link function. Period estimates are presented as prevalence ratios (PR) and birth cohort estimates as cumulative incidence ratios (RR); 95% confidence intervals and *P* values are reported. In a subsample of 37 volunteers (12.29 ± 2.63 years), the fluoride (F) concentration in toenails was measured using the HMDS diffusion method and an ion-specific electrode. Other samples from the local environment such as food, soil, and coal were also collected.

**Results:**

In 274 children, we found that nonsignificant increases between periods (PR = 1.17; 95% CI: 0.89–1.55) were not explained by birth cohort effects. A total of 37.8% of the subsample had a toenail F concentration ≥2 *μ*g F/g. The salty snacks and seasoning had the highest F concentrations among local environmental samples.

**Conclusion:**

In this population with a high DF frequency according to birth cohort and the evaluated period, the study of soil, coal, and food samples indicated a continued F exposure. F concentration found in the toenails shows a moderate F exposure; nearly a third of the children and adolescents exceeded the adopted threshold of 2 *μ*g F/g. It is important to monitor and explore changes in exposure in highly affected population.

## 1. Introduction

Over the last decades, exposure to fluoride (F) has increased in fluorinated and nonfluorinated communities [1, 2]. The critical moment for the development of dental fluorosis (DF) in primary teeth is between 6 and 9 months of age [3]. In permanent dentition, a longer period should be considered, which varies depending on the tooth type and the duration of exposure to F during amelogenesis [1, 4–7].

Children in contemporary societies may be exposed to multiple sources of F at different periods of their lives, and yet the most appropriate period to supply F maintaining a risk-benefit balance is unknown [5, 8].

The large window of susceptibility of DF is well known [1, 5]. However, there is still controversy regarding the variety of F's sources and the presence of risk factors such as climate, altitude, and geographic conditions [4, 7, 9]. DF is a condition, which is more related to chronic F accumulation during the child's growth and development than with an exposure limited to specific critical periods [4, 5, 10].

Epidemiological studies have focused mainly on its prevalence. Although some consider that a certain degree of DF is physiologic and does not harm the population [11, 12], the high prevalence and severity in populations with systemic fluoridation has generated concern in the scientific community. The DF frequency can vary in the same region over time, depending on the behavior of its risk and protective factors. These factors can affect the rates according to the cohort [13].

The study of the DF frequency changes can provide guidelines to analyze the balance between the etiological factors and identify if the rate variations are associated with the birth cohort. Along with the monitoring of exposure to F through biomarkers in endemic regions, this analysis can contribute to identifying specific population behaviors and yet unidentified sources and factors. F can be a safe and effective preventive method when adequately used, and low constant concentrations in the oral fluids may be sufficient. New studies could change paradigms regarding F's dose and risk-benefit balance and contribute to its epidemiological surveillance [4, 9, 14, 15].

The primary aim of this study was to analyze changes in the DF incidence, according to a birth cohort as a possible explanation for the increase in its prevalence in an endemic region of northern Colombia. The secondary aim was to characterize and explore the F exposure in a case series, using toenails as a biomarker, and to evaluate its presence in food snacks, a widely used food seasoning, and some local geological elements.

## 2. Materials and Methods

### 2.1. Study Design

This analytical cross-sectional study of the cohort effect analyzed the DF frequency in two time periods (2015–2018) in a children population of 8–18-year-old from a region in northern Colombia (El Cedro). We characterized the F concentration in toenails as an exposure biomarker in a nested case series.

This study, which was approved by the CES University's Institutional Committee of Ethics of Research in Humans (Medellin, Colombia) (Act No. 110, Code 718), is part of a macroproject aimed to analyze and characterize DF. The participant children and their parents signed an informed assent and informed consent, respectively.

The El Cedro district in northern Colombia is part of a Health Program of Cooking Salt Fluoridation (180–220 F ppm). This endemic DF population has a mean F concentration of 123.6 ± 89.4 in salt and 0.10 F ppm in water [16]. Its 2015 total population was 929 inhabitants (density of 6.15 inhabitants per km^2^). A first clinical evaluation was conducted in 2015, where 187 children aged 8–12 years (*n* = 225) [16]. During a health visit in 2018, a call was made to all students over eight years old (*n* = 490), including children evaluated in 2015. Only 182 children attended. To analyze the changes in frequency, based on the cohort effect in the prevalence of the samples evaluated in 2015–2018, we included a sample of 274 children. All schoolchildren were invited to both examinations.

The inclusion criteria were children residents of El Cedro who were examined in 2015 or 2018 and had at least the maxillary and mandibular first permanent molars and permanent incisors. The exclusion criteria were the presence of syndromes associated with enamel malformations, enamel defects caused by trauma, and children wearing orthodontic appliances.

The clinical examination was performed in a dental office equipped with artificial light, a suction system, and an air-water syringe, using no. 5 mouth mirror and the WHO periodontal probe [17]. After cleaning with a professional prophylaxis and drying the teeth with gauze, two standardized examiners (0.89 and 0.87 to Kappa intra and interexaminer, respectively) on the Thylstrup-Fejerskov index (TFI) [18] for the DF diagnosis performed a complete examination of all permanent teeth present. The diagnosis of white spot lesions and other enamel opacities was based on the Seow criteria [19]. The examiners were the same at both timepoints.

According to the birth year, the participants were classified into cohorts (2000–2002, 2003–2005, 2006–2008, and 2009–2010). The cohort effect was quantified as positive cases in each period divided by the number of children born in the same period. The period effect was calculated as the total number of positive cases divided by the total number evaluated in 2015 and 2018. Also, variables such as age at examination and year were considered.

### 2.2. Sample Collection

#### 2.2.1. Toenails

For the exploration, analysis, and quantification of fluorides, with the aim of achieving a sample size between 30 and 40 samples, toenails were collected from a sample of 37 children with DF diagnostic. The inclusion criteria were children residents of El Cedro, of both genders, with clinical exam, TFI score ≥1, with a preference for a score TFI > 1.

For the toenail collection, parents were instructed to allow children's toenails to grow for 15 days before cutting. They were also asked not to paint the toenails. The collected samples were stored and coded. Each toenail was washed with deionized water using an interdental brush, deionized in a water container for 10 minutes, and dried at 60 ± 5°C for 12 hours. Finally, the toenails were weighted.

#### 2.2.2. Soil and Coal

As an exploratory analysis, soil and coal samples were taken from two strategic areas of the region: one in the central part and another from a peripheral area of El Cedro. A 40 cm hole was made for the soil sampling, collecting its deepest soil and filling two 200 ml containers. The coal sample was taken directly from the woodstove.

#### 2.2.3. Snacks and Food Seasoning

Samples of the children's most commonly consumed snacks and from a popular food seasoning used in this region were obtained from El Cedro schools and nearby stores.

Soil, coal, and food samples were macerated, placed in Petri dishes, and weighted to obtain 0.50–0.59 mg in duplicates. The samples were kept at room temperature until they were analyzed.

### 2.3. Fluoride Analysis

Fluoride analysis was performed in the laboratory at the Bauru School of Dentistry, University of São Paulo, Brazil. All fluoride analysis performed on samples were made in duplicate. Each snack or food was measured twice for fluoride analysis; all the toenails samples were made in biological duplicates. All were encoded and chemically analyzed by diffusion. The toenail samples ranged from 10 mg to 12 mg. [20].

The F concentration was determined after overnight hexamethyldisiloxane-facilitated diffusion [21] as modified by Whitford [7], using a model 9409 F electrode (Orion) and a miniature calomel reference electrode (Accumet, no. 13-620-79) connected to a potentiometer (model EA-940, Orion). During diffusion (at room temperature), the solutions in the nonwettable Petri dishes (Falcon no. 1007) were gently swirled on a rotary shaker. F standards (50.0, 10.0, 5.0, 1.0, 0.5, and 0.25 *μ*g F) were prepared by serial dilution of a stock solution 0.1 M F (Orion) in triplicate and diffused in the same manner as the samples. The mean repeatability of the readings, based on duplicate samples, was 95% (it means that only data with 95% of repeatability on duplicate readings were acceptable). The standard curve had a coefficient of determination ≥0.98 [20].

### 2.4. Statistical Analysis

The data were analyzed in the Software for Statistics and Data Science (STATA), version 16. For the descriptive analysis, we used frequencies and percentages for the categorical variables and measures of central tendency and dispersion for the quantitative variables. For the risk difference analysis, according to the birth cohort, a generalized linear model using the binomial family and logarithmic link considered the age and examination year as covariates. The results were presented as RR with 95% confidence intervals, *P* value, and second-generation *P* values (SGPV) [22].

All the data obtained from the direct F measurement were analyzed and recorded for their description. Variables such as sex and age were dichotomized when analyzing the F concentration in the toenails. For the TFI grade, its highest score was assigned to each child. These variables were compared using the Wilcoxon test (Mann–Whitney) at a significance level of 5%.

## 3. Result

### 3.1. Changes in the DF Frequency according to the Birth Cohort

We included 274 children aged 8–18 years. We found a higher DF prevalence in 2018 (96.6%) than in 2015 (85.1%), but this difference was not significant (*P* > 0.05). The DF frequencies from 2015 to 2018 are shown in Table 1.

During the 2018 evaluation, we identified a higher DF frequency (RP 1.17; 95% CI 0.89–1.55), compared to the 2015 evaluation (SGPV = 0.21). However, this difference cannot be explained by an increase in the DF incidence according to birth cohorts (Table 2).

### 3.2. Fluoride Concentration in Biological and Environmental Samples

We analyzed the F concentration in the toenails of 37 children. The mean age was 12.29 ± 2.63 years, and 54.0% was males. The mean F concentration in toenails was 2.48 ± 2.29 *μ*g F/g (F ppm), with a range value of 0.03–8.68 *μ*g F/g (F ppm). 37.8% of children presented values ≥ 2 *μ*g F/g (F ppm) [23] in the toenails.  Table 3 shows the F concentration by sex, age, and TFI grade.

When comparing the F concentration in toenails according to age, gender, and TFI grade, we found no significant differences in the age and gender distribution, although the F mean concentration in children aged 7–12 years (1.67 ± 1.69 *μ*g F/g) was lower than in children aged 13–18 years (3.09 ± 2.52 *μ*g F/g) (*P*=0.06). The toenails of children with TFI ≤ 3 (*n* = 23) presented a significantly higher F mean concentration (3.17 ± 2.40 *μ*g F/g) compared to children with TFI ≥ 4 (*n* = 14; 1.33 ± 1.56 *μ*g F/g) (*P*=0.015).

Table 4 shows the F concentration in the soil, coal, and food samples. The mean F concentration was found to be very low (0.12 ± 0.146 *μ*g F/g). The samples collected from the central part showed a slightly higher F concentration than those collected from the peripheral area, although the difference was not statistically significant. The mean F concentration found in the snacks (11 snacks-one sweet-soda) and one food-seasoning product was 0.5 ± 0.55 *μ*g F/g (F ppm F). A higher F concentration was found in salty snacks (Almuercito and Crakeñas cookies). Food seasoning showed the highest F concentration (6.7 *μ*g F/g; F ppm).

## 4. Discussion

According to the birth cohort, the change in incidence cannot explain the increase in the DF frequency in El Cedro between 2015 and 2018. Although DF seems less frequent in older children (born 2000–2002), the magnitude is generalized. Although not significant, the difference in gender distribution may suggest a different risk exposure between males and females. Exposure time relates to the increase in the DF incidence among the birth cohorts.

As occurred in the 2006–2008 birth cohorts, which showed DF frequencies of 73.0% (2015) and 98.7% (2018), the variation between the birth cohorts suggests the presence of different associated factors according to the period. Older children (2000–2002) showed the lowest DF frequency in 2018 (88.8%), which might indicate that some mild lesions may become clinically invisible over time, due to wear, leading to lower frequencies [24]. The age at which the clinical examination is performed is crucial to detect the disease clinically [5].

As a secondary analysis, we explored F exposure in a series of DF cases. Nails are a noninvasive, readily available biomarker of chronic, subchronic, and acute F exposure. The F concentration found in the toenails confirms its value in measuring a community's chronic F [23, 25]. The exposure time reflects the increase in the DF incidence in the birth cohorts and coincides with the F concentration found in the study population's toenails. However, the sample size and lack of follow-up of the cohorts are considerable limitations of our study. And also, this study design cannot demonstrate a temporal connection between the F toenail concentration and the DF onset; however, it aligns with the high DF prevalence (96.6%) found in this population. The F concentration shows the F intake level over a long period, which can be useful for DF risk assessment [7, 14, 26]. However, the age group of the studied cases is outside the window of susceptibility, and therefore, age is crucial when predicting or assessing the risk for DF.

Despite being a small sample, the results show a higher DF prevalence than another cohort study in a Mexican population with different exposures according to the domestic salt fluoridation program [13]. Due to the lack of specific incidence studies in Colombian populations, it is challenging to compare our findings with others from similar studies. The DF prevalence found in our study is higher than those found in other populations with drinking water fluoridation, such as the USA. In the US national surveys 1986-1987, 1999–2004, and 2011-2012, the DF prevalence has increased from 22% to 41% and to 65%, respectively [15]. In Colombia, the reported DF prevalence is 11.5% (1998) and 62.1% (2014-2015) [27].

The F concentration found in the studied cases' toenails coincides with that found in similar studies, i.e., toenail samples, age group, and the number of subjects, conducted in other geographic areas [20]. Elekdag-Turk et al. [25] found a mean F concentration of 2.34 ± 0.26 mg/kg in endemic regions and a mean of 0.98 ± 0.08 mg/kg in nonendemic regions. Buzalaf et al. [20] and Amaral et al. [28] reported F concentrations of 1.49–2.80 mg/kg and 2.15 (0.39)–2.64 (0.41) *μ*g F/g, respectively.

Our findings differ from other studies that used toenail samples. Buzalaf et al. [23] found a higher mean F concentration (4.22 (2.45) *μ*g F/g). Similarly, in regions with low F in water, Idowu et al. [26] found F concentrations of 3.237 (2.636) and 3.378 (2.197) *μ*g F/g in fingernails and toenails, respectively. Also, the F concentration found in our study population is very low compared to areas with higher F in water, where F concentrations in nails of 10.420 (3.761) and 10.371(3.907) *μ*g F/g have been reported [26].

Although some studies have reported higher F concentrations in fingernails than in toenails, it is currently recommended to use toenail samples, especially from the first toe, because it is fast growing and provides enough sample for the analysis. Some reports have shown that toenails are less exposed to external contamination than fingernails [7, 20, 25, 28]. However, this can vary depending on the lifestyle of the rural populations.

When correlating the F concentration in toenails with age and DF severity according to the TFI [18], we found a higher concentration in the group aged 13–18 years, which may indicate a higher exposure over time [5], and also, it could indicate differences in the F absorption after tooth development. In contrast to other studies that showed that F concentration in nails increases with the DF severity [14, 23], we found a higher mean F concentration in children and adolescents with TFI ≤ 3. This divergence may indicate an altered F absorption in the study population, possibly due to genetics variations, epigenetic factors, malnutrition, diet composition in terms of protein, carbohydrates and fat, and intake of micronutrients such as calcium [1, 14, 29, 30].

Although El Cedro is considered an endemic region, its primary source of F exposure is unclear. The analyzed samples showed significant F concentration variability, with salty snacks and food seasoning presenting the highest concentrations. These findings concur with those from other studies, in which there is no direct correlation between the various analyzed sources, F concentration, and DF severity in endemic regions [16, 26, 31]. In Colombia, F concentration in salt has also shown a high variability and sometimes may exceed the maximum permitted level [16, 32]. Each sample was measured twice for fluoride analysis, and the duplicate samples analysis has been used in different methods [33, 34]. The F concentration found in this study in snacks and food-seasoning is similar to other F concentrations reported by Zohouri and Rugg-Gunn, for cereals (0.2–03 *μ*g F/g), mixtures of spice (2.41 *μ*g F/g), and chilli powder (1.82 *μ*g F/g) [33]. The difference in fluoride content that may appear in different snacks and food-seasoning lots is worthless, considering that the major source of fluoride on Earth provides from soil (rocks) and thereafter on salt. Assuming that package is manufactured on the same place with the same raw material, variations in parts per million (ppm) are despicable. Although the soil and coal samples were taken from only two places and thus not represent a probabilistic sample from various geographical areas, their low F concentration indicates its presence in the community environment.

Our findings regarding F concentration reflect a simple exploratory analyze. However, the lack of research on nails as an F biomarker and analysis of different elements in communities with salt fluoridation makes this study contribute to the knowledge of nails as a biomarker of F exposure and the DF frequency in low altitude, warm-humid climate endemic regions. There is a need for studies with a similar approach involving larger samples and considering DF window of susceptibility, i.e., 3 months to 8 years old [5, 20]. Future studies conducted in this region should also consider other factors such as the increase in mining exploitation and access to dental services (last decade), diet, and salt intake, in order to contribute to the development of social and health policies.

The recommendations for F use should be based on the evidence of the risks and benefits for each population [11]. Actions such as those implemented in recent years in the USA and Malaysia, where F concentration in water was lowered to 0.7 mg/L [15] and 0.5–0.7 ppm [35], respectively, can be adopted by other countries with fluoridated salt and endemic regions with fluorosis, such as Colombia. The study of DF in endemic regions requires a more in-depth analysis to understand its etiological, epidemiological, and biological contexts. DF prevalence is increasing in this population as in the world; DF and the F concentrations are related, but its clinical manifestation and variations in the populations are multifactorial.

## 5. Conclusion

In this population with a high DF frequency according to the birth cohort and evaluated period, the study of soil, coal, and food samples indicates a continued F exposure. F concentration found in the toenails shows a moderate F exposure; nearly a third of the children and adolescents exceeded the adopted threshold of 2 *μ*g F/g. It is important to monitor and explore changes in exposure in highly affected population.

## Figures and Tables

**Table 1:** Distribution of the analyzed variables in the study children, 2015 and 2018.

Variable	Cases	*n*	%
Examination year			
2015	80	94	85.1
2018	177	180	96.6

Birth of cohort			
2000–2002	8	9	88.8
2003–2005	116	121	95.8
2006–2008	103	114	90.3
2009–2011	30	30	100

Age (years)			
8-9	57	67	85
10–12	129	134	96.2
13–15	63	64	98.4
16–18	8	9	88.8

**Table 2:** Examination year and cohort analysis of the DF presence in the study children, 2015 and 2018.

	Ratio	95% CI	*P* value	SGPV^*∗*^
Examination year				
2015	1.00			
2018	1.17	0.89, 1.55	0.261	0.21
Birth cohort				
2000–2002	0.89	0.41, 1.94	0.767	0.574
2003–2005	1.03	0.68, 1.57	0.889	0.461
2006–2008	0.95	0.63, 1.44	0.804	0.559
2009–2011	1.00			

^*∗*^Second-generation *P* values.

**Table 3:** Fluoride concentration in the toenails by gender, age, and TFI score of the study children, 2018.

Variable	*n* (%)	*μ*g F/g^*∗*^	*P* values
Gender			
Male	20 (54.0)	2.51 ± 2.23	0.989
Female	17 (46.0)	2.44 ± 2.25	
Age (years)			
8–12	16 (43.3)	1.67 ± 1.69	0.060
13–18	21 (56.7)	3.09 ± 2.52	
TFI			
TF ≤ 3	23 (62.2)	3.17 ± 2.40	0.015
TF ≥ 4	14 (37.8)	1.33 ± 1.56	

^*∗*^Mean (SD) *F* = *μ*g F/g (two measurements).

**Table 4:** Fluoride concentration in soil, coal, snacks, and food seasoning samples, 2018.

*Sample*	*μ*g F/g
Soil central part	0.30
Coal ash central part	0.20
Soil peripheral part	0.01
Coal ash peripheral part	0.00

*Snacks*	
Jell-pep strawberry milk	0.10
Candy punch	0.10
Colored miniatures	0.10
Sweet soda^*∗*^	0.20
Milo cookies	0.30
Bubbaloo gum-strawberry-grape	0.30
Festival strawberry cookies	0.40
Bubbaloo gum-mango	0.40
Milk cookies	0.50
Mint candy	0.60
Almuercito	**1.00**
Crakeñas cookies	**2.00**

*Food seasoning*	**6.70**

^*∗*^ *μ*g F/ml.

## Data Availability

The data used to support the findings of this study are available from the corresponding author upon request.
